# Molecular Features Contributing to Virus-Independent Intracellular Localization and Dynamic Behavior of the Herpesvirus Transport Protein U_S_9

**DOI:** 10.1371/journal.pone.0104634

**Published:** 2014-08-18

**Authors:** Manuela Pedrazzi, Bradley Nash, Olimpia Meucci, Renato Brandimarti

**Affiliations:** 1 Department of Pharmacy and Biotechnology, University of Bologna, Bologna, Italy; 2 Department of Pharmacology and Physiology, Drexel University College of Medicine, Philadelphia, Pennsylvania, United States of America; 3 Department of Microbiology and Immunology, Drexel University College of Medicine, Philadelphia, Pennsylvania, United States of America; International Centre for Genetic Engineering and Biotechnology, Italy

## Abstract

Reaching the right destination is of vital importance for molecules, proteins, organelles, and cargoes. Thus, intracellular traffic is continuously controlled and regulated by several proteins taking part in the process. Viruses exploit this machinery, and viral proteins regulating intracellular transport have been identified as they represent valuable tools to understand and possibly direct molecules targeting and delivery. Deciphering the molecular features of viral proteins contributing to (or determining) this dynamic phenotype can eventually lead to a virus-independent approach to control cellular transport and delivery. From this virus-independent perspective we looked at U_S_9, a virion component of Herpes Simplex Virus involved in anterograde transport of the virus inside neurons of the infected host. As the natural cargo of U_S_9-related vesicles is the virus (or its parts), defining its autonomous, virus-independent role in vesicles transport represents a prerequisite to make U_S_9 a valuable molecular tool to study and possibly direct cellular transport. To assess the extent of this autonomous role in vesicles transport, we analyzed U_S_9 behavior in the absence of viral infection. Based on our studies, Us9 behavior appears similar in different cell types; however, as expected, the data we obtained in neurons best represent the virus-independent properties of U_S_9. In these primary cells, transfected U_S_9 mostly recapitulates the behavior of U_S_9 expressed from the viral genome. Additionally, ablation of two major phosphorylation sites (i.e. Y_32_Y_33_ and S_34_ES_36_) have no effect on protein incorporation on vesicles and on its localization on both proximal and distal regions of the cells. These results support the idea that, while U_S_9 post-translational modification may be important to regulate cargo loading and, consequently, virion export and delivery, no additional viral functions are required for U_S_9 role in intracellular transport.

## Introduction

Cellular functionality heavily relies on efficient transport of individual components, from single molecules to entire organelles. Even under resting conditions intracellular traffic of these various cellular entities is significant and finely regulated; this is achieved through the activities of specialized molecular motors that move cargoes along cytoskeletal structures [1], [2]. The microtubular cytoskeleton offers a great backbone to travel in every direction, from the cell center to the periphery and viceversa. Transport occurring in the same direction as microtubules polymerization, which has been designated “+”, is referred to as anterograde transport, and is usually directed toward the periphery of the cell. Conversely, motion toward microtubules “−” end is defined retrograde, and mostly points to the nucleus [3].

Amongst many other cellular components, special and unwanted cargos are represented by viruses, especially those, like retroviruses and herpesviruses, which must reach the nucleus to complete their replication cycles. As widely acknowledged, viruses are able to efficiently exploit physiological functions (e.g. S phase induction [4]; blockage of MHC-I maturation [5], [6]) through the activity of specialized proteins that specifically target cellular factors. Conversely, viral proteins may be seen as tools to both decipher cellular functions and re-program them for different purposes.

U_S_9 is a gene well conserved in the family of alphaherpesviruses, the group of herpesviruses characterized by the ability to establish a lifelong latent infection in the peripheral nervous system of their host. It encodes a small tail-anchored, type II membrane protein. For a long time neglected as a non essential viral product, U_S_9 has recently gained more attention because viruses deleted in the U_S_9 gene show defects in the ability to move in the anterograde direction in the axons and to establish secondary infections in the brains of infected animals [7]–[12]. Virus anterograde transport occurs inside vesicles; besides being a constitutive component of transported virions, U_S_9 is present on transport vesicles membrane. The impairment shown by deletion viruses implies that U_S_9 participates in the process of virus egress, and that this effect is dependent on the ability of the protein to directly or indirectly regulate the interaction of the viral particle (or of its parts) with the transport machinery. In Pseudo-Rabies Virus (PRV) the role in transport played by U_S_9 has been extensively investigated [7], [12]–[18] and recently visually demonstrated *in vivo* by Taylor et al. [19]. However, beside the U_S_9 activity related to virus replication and diffusion in the infected host, it is of great interest to understand the molecular features that confer the protein its ability to drive vesicles transport. Localization studies showed that U_S_9 mostly (but not exclusively) accumulates in Trans Golgi Network [8], [20], but it is also detected in more peripheral regions of the cell, as well at the plasma membrane, in agreement to the assigned transport task deduced from deletion studies.

Starting from the acknowledged role played by U_S_9 in virus transport, we decided to look at U_S_9 *stand alone* properties using GFP-tagged constructs in a virus-free cellular environment, both in fixed cells and in real time experiments, aimed to investigate if and to which extent U_S_9 properties that in the viral context serve virus transport and infection spread, can be directly ascribed to U_S_9. The results presented here highlight the dynamic behavior of U_S_9, supporting the idea that the viral protein is able to autonomously interact with the cellular transport machinery, in a cell type independent manner. In fact, U_S_9 is always detectable in both proximal and peripheral cellular regions, as well as on the plasma membrane. This ability, which in the viral context supports transport and delivery of viral particles or of its components, is maintained even in the absence of other viral factors. By using truncated forms of the viral protein, we observed that while the U_S_9 trans-membrane domain partially recapitulates the functional behavior of the full length protein, as it is still able to dictate localization of the fused GFP moiety in both cytosolic puncta and at the plasma membrane, it clearly differs from the full length GFP-U_S_9. Notably, the major difference ascribed to the absence of the U_S_9 cytosolic domain is the reduced punctuate staining and the enhanced accumulation of the protein at the plasma membrane. Finally, mutagenesis of key tyrosines and serines in the acidic domain located in the middle of the cytosolic portion of the protein and important for virus transport do not affect U_S_9 behavior, supporting the hypothesis that post-translational modifications represent a tuning system adopted by the virus to regulate cargo loading and, consequently, virion export and delivery; however, and most importantly, they are not required for U_S_9 transport inside the cell.

## Results

### Construction and analysis of GFP-U_S_9 fusion proteins

**Figure 1:**
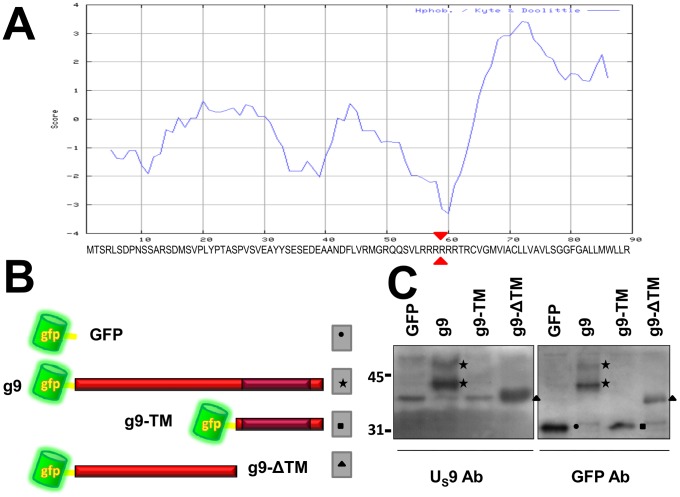
Construction and analysis of GFP-U_S_9 chimeras. A) Kyte-Doolittle hydropathicity profile of U_S_9 aminoacid sequence (aa reported under the plot). B) schematic topological orientations of GFP-U_S_9 fusion proteins. The darker area inside the red ribbon, corresponding to the U_S_9 full length sequence, indicates the position and the approximate extension of the TM domain. The two truncated chimeric U_S_9 forms g9-ΔTM and g9-TM were generated by splitting the U_S_9 aa sequence in the middle of the 6 arginine motif, as pointed by the arrowheads in the sequence in A. C) Western blot analysis of GFP-U_S_9 fusion proteins expressed in transfected cells. Two identical membranes containing lanes with proteins from MDA cells transfected with the indicated constructs and separated on 12% polyacrylamide gels were incubated with U_S_9 and GFP antibodies. Closed symbols indicate bands corresponding to the different fusion proteins (as schematically indicated in B). M*_r_* in kDa ×10^3^ is reported on the left.

The U_S_9 gene in the HSV genome encodes a protein of 90 aminoacids [21], highly conserved amongst alphaherpesviruses, with an essential role in anterograde axonal transport [7]–[12], [22]–[23]. As predicted by its hydropathicity profile (figure 1A), U_S_9 has been shown to be a tail-anchored type II membrane protein, with the N-terminal domain hanging in the tegument region comprised between virion envelope and nucleocapsid [24]–[25]. Here we studied the U_S_9 transport properties by tagging the protein with GFP. The U_S_9 gene was cloned into pEGFP-C1, to create the GFP- U_S_9 chimera (figure 1B) named g9. Two more plasmids were constructed, with the same topological organization but with two complementary truncations of the U_S_9 protein (as indicated by red triangles in the sequence in figure 1A): g9-TM (GFP-U_S_9 Trans-Membrane) and g9-ΔTM (GFP-U_S_9 ΔTrans-Membrane), encoding GFP fused to the U_S_9 hydrophobic and hydrophilic domains, respectively (figure 1B). The three constructs, along with the parental plasmid pEGFP-C1, were transfected into MDA cells, and protein contents were analyzed by Western blot using antibodies directed against U_S_9 and GFP (figure 1C). As expected, a band with a relative size corresponding to the GFP proper molecular weight was recognized by the GFP antibody in the GFP only lane (right panel), while no bands in the same region of the gel could be detected with the U_S_9 Ab. Cells transfected with g9 plasmid expressed a protein specifically reacting with U_S_9 and GFP Abs. Both Abs detected a major band of about 42 KDa (corresponding to the expected molecular mass for the fusion protein), and a higher minor band, likely the result of post-translational modifications – in line with reports from infected cells where U_S_9 typically shows a complex migration pattern partially dependent on enzymatic activity [26]–[30].

**Figure 2:**
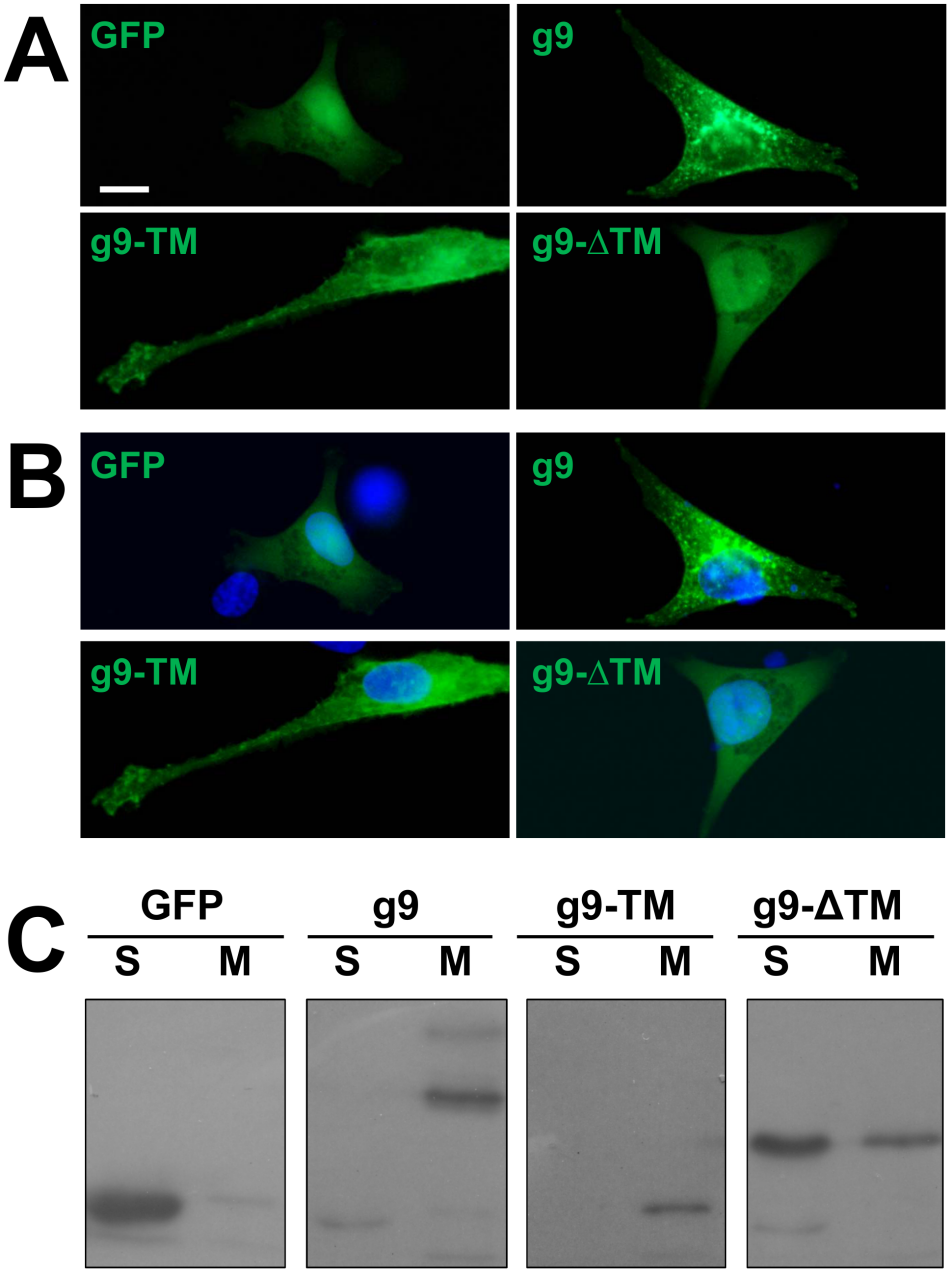
Intracellular localization and membrane association of U_S_9. A) MDA cells transfected with GFP, g9, g9-TM (GFP fused the U_S_9 trans-membrane domain), and g9-ΔTM (GFP fused the U_S_9 hydrophilic domain) were imaged under a fluorescence microscope. Nuclei were stained with Hoechst 33342 (blue) and images were merged in B). C) Proteins extracted from MDA cells expressing the same constructs described in A) were divided in post-nuclear soluble (S), and membrane (M) fractions, separated on SDS Page, and revealed with GFP antibody. Bar = 10 µm.

The g9-TM chimeric protein could be detected only with the GFP Ab, since the U_S_9 Ab is directed against the U_S_9 hydrophilic domain that is missing in the fusion protein [31]. The migration pattern of g9-TM appears very similar to that of GFP, due to the presence of a relatively long extra peptide originated by the translated multiple cloning site sequence in pEGFP-C1. As a consequence, peptides encoded by pEGFP-C1 and g9-TM differ for just 12 amino acids in length. We validated all our constructs by sequencing; more importantly, additional experiments (shown in figure 2 and discussed later) also address this issue and undoubtedly confirm the correctness of the g9-TM construct and the U_S_9 trans-membrane domain contribution to U_S_9 localization and transport properties. Removal of TM domain from U_S_9 in the g9-ΔTM plasmid results in the lack of 32 aa from the fusion protein, and the bands present in both panels in figure 1C (lanes g9-ΔTM) migrated as expected (Mr of about 38 KDa).

### Localization of U_S_9

Given its role in viral particles transport, the cellular compartments in which U_S_9 accumulates and the contributions of U_S_9 hydrophilic/hydrophobic domains to its localization have been widely studied. Early experiments with PRV U_S_9 and its homologs from other alphaherpesviruses demonstrated that U_S_9 mainly accumulates to the Trans Golgi Network (TGN) but it is also detected in distal intracellular regions as well at the plasma membrane [24]–[25], which is in agreement with its role in virus transport [7]–[15], [20], [26]. These studies utilized specific antibodies against the viral protein and Hemo-Agglutinin or GFP tags. Notably, in the context of viral infection, GFP-U_S_9 can functionally substitute wild type U_S_9, and has been used to trace protein localization in infected cells [19].

Here, g9, g9-ΔTM, g9-TM, or the control parental plasmid pEGFP-C1, were transfected into MDA cells, and then imaged 16 hours post transfection. In the absence of specific localization signals, GFP expression yielded a diffuse fluorescent staining in both cytoplasm and nucleus (figure 2A–B). Expression of g9 was strongly associated with a bright punctuate pattern (figure 2A–B). Fluorescent signals were completely excluded from nuclei of transfected cells, and widely distributed into the cytoplasm, in discrete puncta. The punctuate staining is typical of vesicles, as expected for a membrane protein involved in intracellular transport and as already reported [8], [12], [15], [24], [25]. Puncta were detectable throughout the cytoplasm, and a weak signal was also associated with the plasma membrane. U_S_9 accumulation in the central region of the cell has been associated with markers of the Trans Golgi Network, the putative site of virions assembly and sorting [8], [15]–[16], and this region has been defined as the steady-state site of U_S_9 localization. In all our experiments, this fluorescent pattern was always reproduced, with only the intensity of the fluorescent signal in the perinuclear region possibly diverging. However, not to interfere with physiological variability, we did not attempt to establish stably transfected cell clones, and always performed transient transfections. Nevertheless, labeled vesicles in distal regions were always detected, even in cells with a heavy perinuclear fluorescent signal.

Cells transfected with g9-TM (figure 2A–B) showed a comparable yet clearly distinguishable fluorescent pattern, comprising both intracellular punctuate staining and plasma membrane fluorescence. Indeed, g9-TM labeling was more pronounced at the plasma membrane, as compared to the full length protein, while intra-cellular punctuate signal was reduced. These data suggest that the 32 aa long trans-membrane domain of U_S_9 is sufficient to drive the insertion of the GFP molecule (which is almost 3 and 8 times larger than U_S_9 and U_S_9-TM, respectively) into the same vesicles already described for the full length protein; however, in the absence of the cytosolic domain, the fusion protein accumulates at the plasma membrane. On the other hand, as expected, removing the U_S_9 trans-membrane domain from the fusion protein dramatically changes the fluorescent pattern. Cells transfected with g9-ΔTM appeared undistinguishable from cells expressing GFP alone, as the staining was homogenously distributed throughout the cytoplasm and the nucleus. These results clearly confirm that the association of U_S_9 with the vesicular pattern revealed by the fluorescent punctuate labeling depends on the presence of its trans-membrane domain. Moreover, the differences in localization between g9 and g9-TM suggest a role for the U_S_9 cytosolic domain in regulation of protein export to the plasma membrane. In addition to the U_S_9 fluorescent fusion proteins described above, we also generated a U_S_9 chimera with GFP fused to the C-terminal portion of U_S_9, and named it 9 g. When transfected in MDA cells, the fluorescent pattern of 9 g was undistinguishable from that of g9 (not shown). In this study we opted to use g9 since its GFP moiety has a predictable minor impact on the U_S_9 structure and previous studies showed that 9g does not fully reproduce the behavior of wild type U_S_9 [32].

To further investigate the biochemical properties that confer U_S_9 the described intracellular punctuate distribution, we analyzed the presence of the chimeras in the cytosolic and the membrane fractions [33], [34]. The electrophoretic profile of proteins transiently expressed from GFP, g9, g9-TM, and g9-ΔTM plasmids and separated into post-nuclear, cytosolic soluble (S) and membrane (M) fractions is shown in figure 2C. GFP was easily extracted in the soluble (S) fraction and predominantly present in the S lane in figure 2C. On the other hand, g9 protein was detectable in the membrane (M) lane, where membrane-associated proteins accumulate, as predicted by the presence of a trans-membrane domain at its C-terminus. A similar partition was seen with g9-TM, again detected in the M lane, indicating that the fluorescent pattern in figure 2A–B is dependent on the ability of U_S_9 TM domain to link GFP to vesicles and plasma membranes. This result also confirmed the correctness of the g9-TM construct, as it clearly differentiates it from GFP in spite of their similar migration pattern shown in figure 1C (lanes GFP and g9-TM in the right panel). In cells transfected with g9-ΔTM, the band recognized by the GFP Ab was mainly present in the S lane, though the same band was also detectable, at a lower level, in the M lane. Results presented here are representative of several experiments, and were reproduced with a different fractionation protocol (not shown). We do not know how significant is the appearance of the g9-ΔTM band in the membrane-related lane, but decided not to further investigate this point, as the relative amount of the g9-ΔTM band in the S lane was higher than in the M lane, and its fluorescent localization was strikingly different from those of g9 and g9-TM and undistinguishable from that of GFP.

### U_S_9 localization is cell type independent

**Figure 3:**
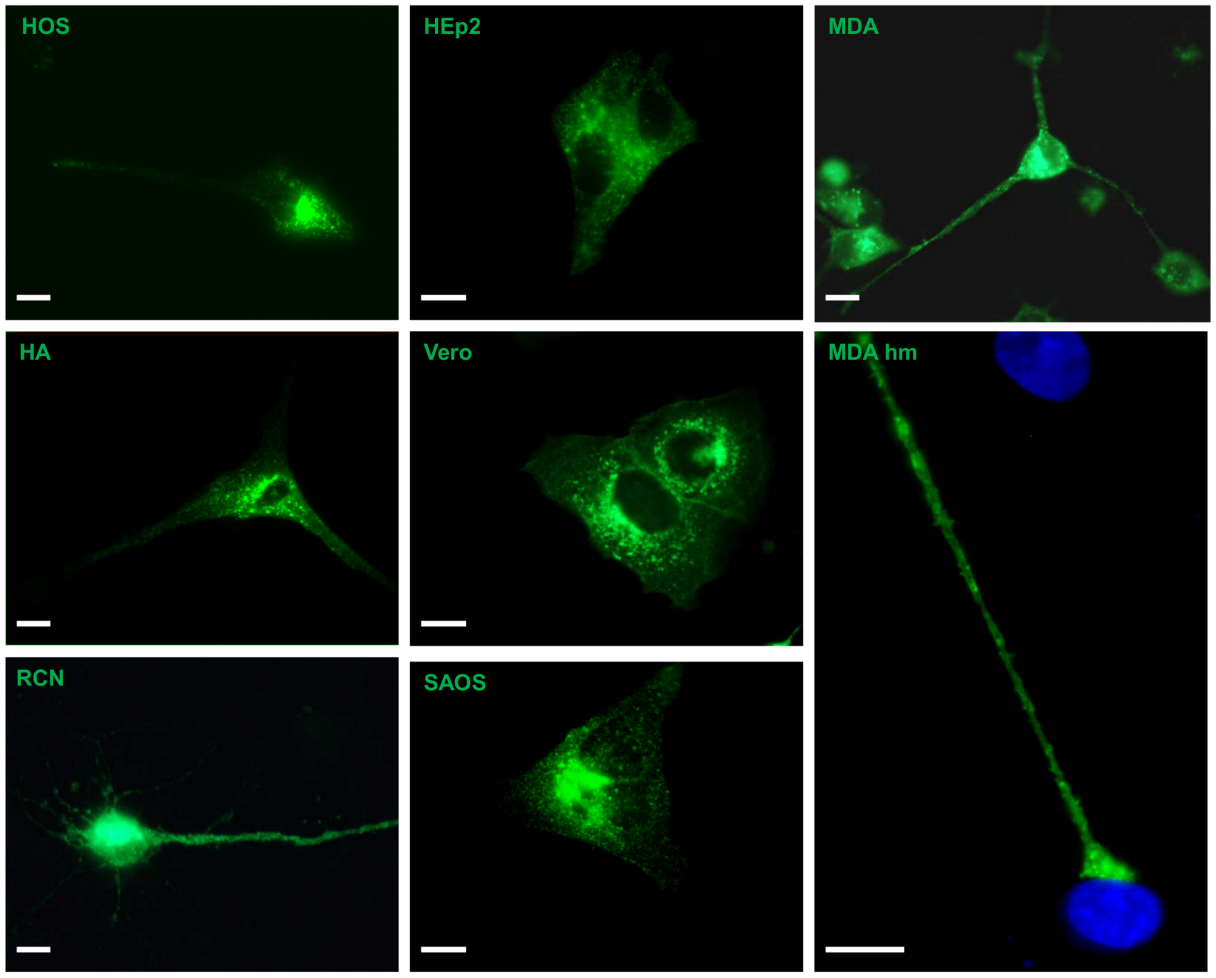
GFP-U_S_9 localization. Cells shown here are representative of several experiments and all share the typical GFP-U_S_9 pattern, i.e. a bright punctuate signal present throughout the cytoplasm and partially concentrated in a peri-nuclear region. Additionally, GFP-U_S_9 can be seen at the plasma membrane, while no fluorescent signal is present in the nuclear region. In the panel labeled MDA hm (higher magnification), the microphotograph focuses on the extension tip protruding from one transfected cell and reaching the body of one untransfected cell. GFP-U_S_9 can be detected in vesicles present in this distal region as well as on the plasma membrane. In green: GFP-U_S_9 fluorescence; nuclei are stained in blue with Hoechst 33342. Bar = 10 µm.

In this set of experiments, meant to investigate U_S_9 ability to maintain its typical localization pattern independently from other viral factors, we introduced the g9 plasmid, carrying the full length gene fused to GFP, into several cell types derived from different species, including rat primary neurons (RN) and cells of human origin. All cells were transiently transfected and fixed 16 hours post transfection. Images representative of the localization patterns for 7 cell types, HOS, MDA, HEp-2, Saos, Vero, HA, and RN, have been chosen and assembled in figure 3. As already shown in figure 2 for MDA cells, U_S_9-associated fluorescent signals were completely excluded from nuclei of transfected cells, and widely distributed into the cytoplasm, in bright puncta in all cell types tested. Taking in account the morphological differences between these cells, the punctuate staining was similar in all cell types, with a more dense distribution in a perinuclear region, which has been previously defined as the U_S_9 steady-state residence and well characterized with the aid of specific TGN markers [8]. Labeled vesicles in distal regions were always detected, even in cells with a heavy perinuclear fluorescent signal, as clearly seen in figure 3. While the steady-state accumulation of U_S_9 at the TGN is not informative about the behavior of distal vesicles and putative U_S_9 transport properties, the presence of a comparable punctuate pattern in all g9-transfected cells suggests that this feature is solely dictated by U_S_9 sequence, supporting our hypothesis of an autonomous, *stand alone* transport property of U_S_9.

This peculiar trait (i.e. distal localization) of U_S_9 phenotype is better visualized in cells characterized by long protrusions. This is obviously the case of neurons (figure 3-RN), the cell type in which the U_S_9 virus transport function becomes critical. From an experimentally relevant viewpoint, a clear distal staining can be seen in MDA cells. As shown in figure 3 (MDA hm), in g9-transfected MDA cells, the typical punctuate fluorescent staining is detectable in long cellular protrusions and accumulates in large plates contacting neighbor cells.

### Dynamic properties of U_S_9

**Figure 4:**
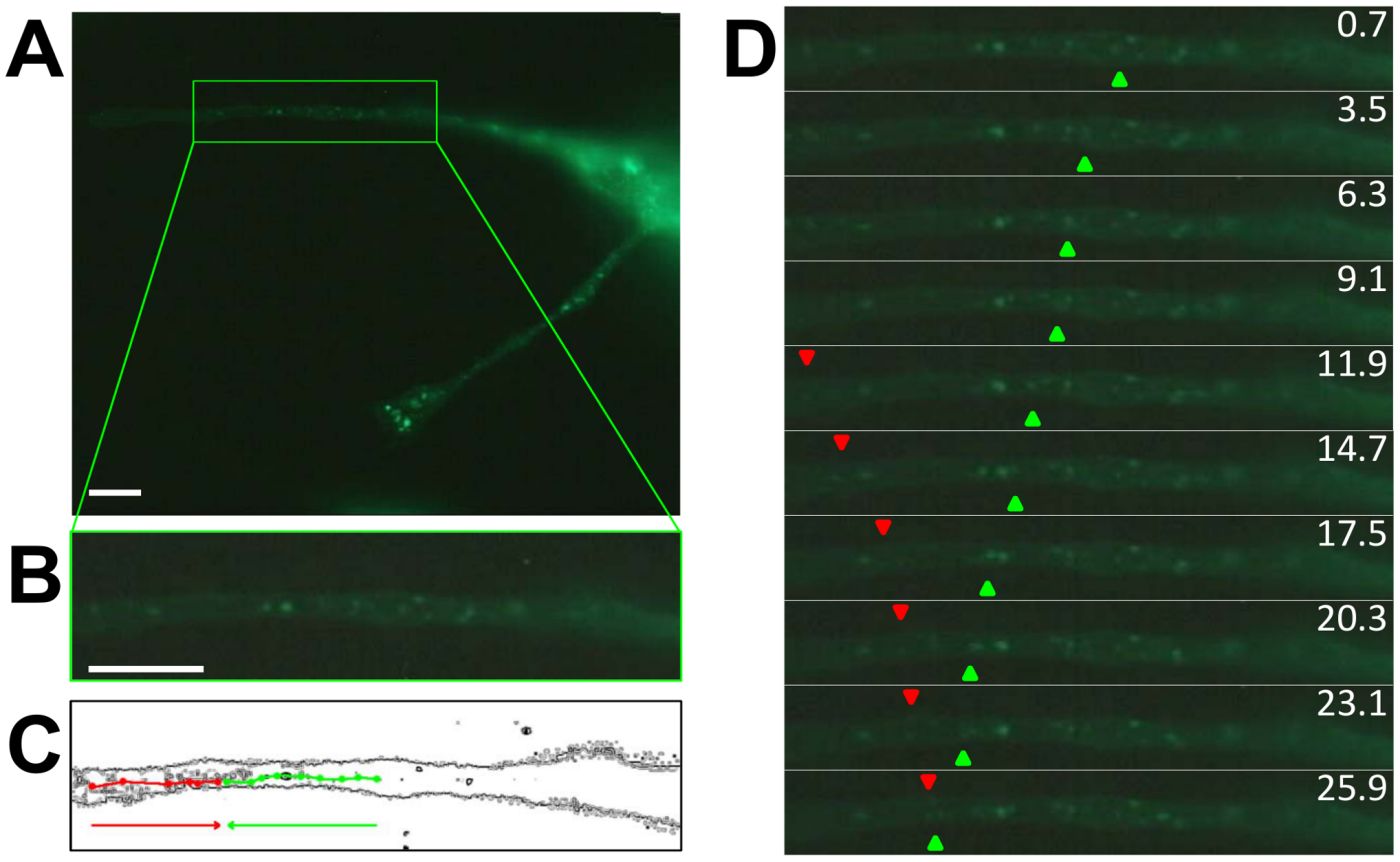
U_S_9 real time imaging. MDA cells were transfected with g9 and then imaged *in vivo* under a fluorescence microscope. In A), the complete field containing a transfected cell with a green boxed area focusing on the first frame (enlarged in B) of movie S1 available online. In C), edges of the same cell portion chosen in B) are shown, with the trajectories of two U_S_9 containing vesicles reconstructed. The two trajectories refer to vesicles moving in the anterograde (green) or retrograde (red) direction. In D), individual frames were aligned, with the green and red triangles indicating the positions of the two vesicles whose trajectories were represented in C), respectively moving in anterograde and retrograde directions (movie S2). Bar = 10 µm.

Data accumulated in PRV and HSV U_S_9 studies showed that U_S_9 is involved in virus transport inside the cells [7], [12], [35], and deletion mutants are defective in anterograde-dependent infection spread [10], [11], [13], [15], [19], [36]. As expected, U_S_9 localization strictly depends on the integrity of microtubular cytoskeleton (Figure S1). We were interested in understanding the dynamic properties of U_S_9 in the absence of other viral factors. Therefore, we imaged the transfected live cells to look at the localization of the protein *in vivo*. MDA cells were transfected with g9, and imaged 16 h post-transfection. The results of these experiments are shown in figure 4 as well as in movies S1 and S2 (Supplemental Online Material). These real time imaging studies confirmed the typical punctuate staining seen in g9-transfected cells (Figure 4A). The green box in the figure frames the region of the cell (figure 4B) that has been followed over time. Selected frames from the complete movie S1 have been arranged in figure 4D and clearly show the dynamic properties of g9, with labeled puncta moving in the cytosol of expressing cells. Some movements appeared to be short in distance, with no preferential direction. However, some fluorescent spots did travel long distances, and those movements align with the longitudinal axis of the analyzed cell protrusion. The series of panels in figure 4D are sequential frames of g9 transfected cells in which two arbitrary chosen vesicles have been tracked with the aid of green and red triangles (Movie S2). The images clearly show long traits of linear movements of g9 containing vesicles. Different labeled vesicles move in anterograde (green triangles) or retrograde (red triangles) motions, and some even appear to change the direction of movement over time. Figure 4C reconstructs the trajectories of the two vesicles described above. The time frame for this dataset was less than 30 seconds, but we do not intend to draw any conclusion about the possible speed of U_S_9 vesicles, as in the many movies we shot there was always a great variability in both speed and direction. Nevertheless, these results undoubtedly indicate that U_S_9 can associate with vesicles transported inside the cell, even in the absence of other viral factors or other viral-dependent modifications, and that its sequence is sufficient to target GFP to the same transport vesicles.

### Transfected U_S_9 recapitulates viral U_S_9 behavior

**Figure 5:**
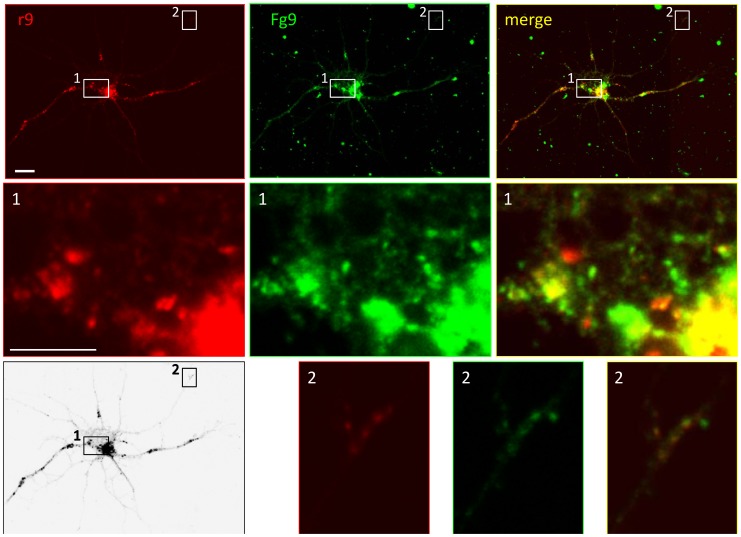
Colocalization of transfected and virus-expressed U_S_9. RNs were transfected with U_S_9 fused to red fluorescent protein (r9) and infected with recombinant HSV-1(F) Fg9, expressing U_S_9 fused to green fluorescent protein (MOI = 10 pfu/cell). One representative neuron is shown here (top row), imaged with a confocal fluorescence microscope to show localization of transfected (red – left panels) and virus-expressed (green – middle panels) U_S_9. Top right panel contains merged images, showing a substantial but not complete colocalization. Central (frame 1) and peripheral (frame 2) regions of the cell are shown at higher magnification in rows 2 and 3, respectively. Both in cell body and at neurite ends, transfected and virus-expressed U_S_9 mostly but not completely colocalize (yellow framed merged images in right panels). Left bottom panel shows black & white rendering of the infected-transfected neuron and is included to help locate the magnified regions in the complex neurons morphology. Bar = 10 µm (5 µm in the magnified panels).

**Figure 6:**
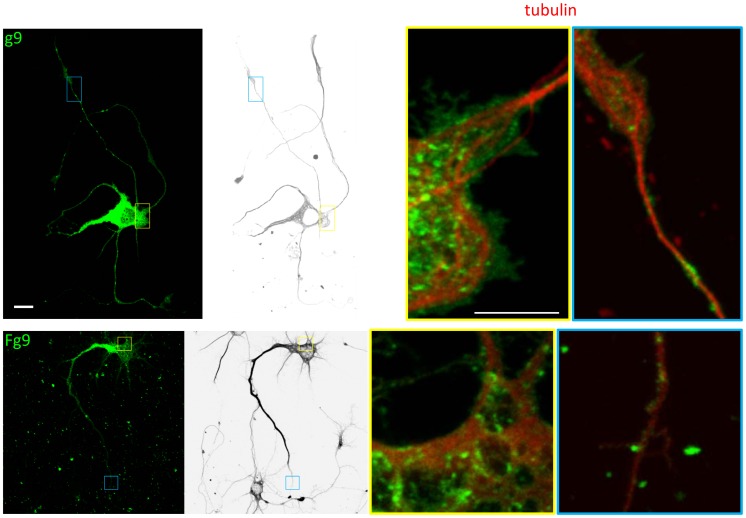
Localization in neurons of U_S_9 expressed from the viral genome or in virus-free context. RNs were either transfected with the GFP-U_S_9 plasmid (g9: top panels) or infected (MOI = 10 pfu/cell) with the recombinant HSV-1(F) carrying the chimeric GFP-U_S_9 gene (Fg9: bottom panels). Left first panels in both rows show the fluorescent pattern of transfected or infected cells, with the bright punctuate signal concentrated in the cell body and also dispersed along neurites. Two representative regions (indicated by small frames in left panels) from both transfected and infected cells were chosen and are shown at higher magnification in the right 2 panels (yellow frames: cell body; blue frames: neurites tips). Black & white rendering of the transfected or infected neurons shown on left panels were obtained by pseudo-coloring the microtubulin cytoskeleton revealed by immunofluorescence (shown in red in the right two panels), and are included to help locate the magnified regions in the complex neurons morphology. Both central and peripheral regions of transfected and infected cells show the microtubular architecture (red) with U_S_9-vesicles (green). In both regions, localization of transfected U_S_9 seems to mostly overlap that of U_S_9 expressed in the viral context. Bar = 10 µm (in the left 2 panels); Bar = 5 µm (in the right 2 panels).

**Figure 7:**
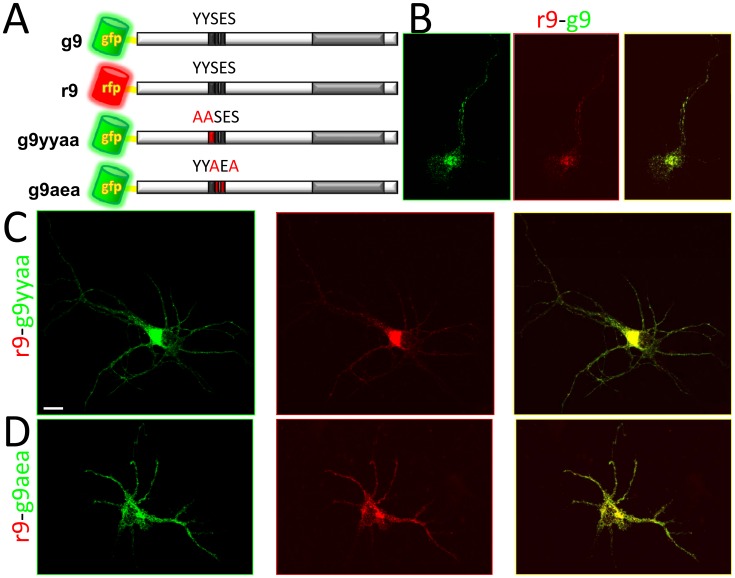
Localization of U_S_9 mutated forms in RNs. A) Two major phosphorylation sites were mutated in the U_S_9 sequence and the modified forms were expressed as fusions with GFP, in the same topological orientation as GFP-U_S_9. r9 and g9 fusion proteins have a completely overlapping staining pattern, as shown in B (right panel is the merge of g9 and r9 images shown on the left). A perfect colocalization is obtained in cells cotransfected with r9-g9yyaa (C) and with r9-g9aea (D). In the figure, GFP- and RFP-chimeras are in green- and red-framed images, respectively, and merged images are framed in yellow in the right panels. Bar = 10 µm.

U_S_9 role in vesicles transport has been extensively investigated with the intent to define its contribution to virus transport. Thus it is now established that U_S_9 is necessary for virus anterograde transport in axons of infected neurons [7], [9]–[12], [15], [22], [35]; likewise, related virus-dependent post translational modifications of U_S_9 have been identified [18], [20], [26], [27]. However, from our perspective (i.e. determining the autonomous ability of U_S_9 to act as a transport protein) it was important to compare the behavior of GFP-U_S_9 expressed from a transfected plasmid to the one present in the context of virus infection, in distal regions of the cell. While TGN resident proteins have been used to positively ascertain the identity of the central region in which U_S_9 accumulates [8], no unambiguous markers of distal vesicles have been identified. Early (Rab4, Rab5) and late (Rab7) endosomal markers showed little colocalization with viral transport vesicles [37]; in other studies, clathrin-like coats were only occasionally associated with L-particle and virion assembly sites [38]. In order to avoid any uneven estimation of the correspondence between *stand alone* and viral U_S_9-labeled pattern, we employed a different approach and decided to use viral U_S_9 as specific marker of these peripheral vesicles. However, this kind of analysis is constrained by the way viruses replicate and assemble, as explained below, and for this reason our comparison strategy included both a direct and an indirect analysis, whose results are shown in figures 5 and 6. For these studies, we replaced the GFP sequence in g9 with the gene coding a red fluorescent protein (RFP), and obtained a new construct, named r9, whose fluorescent distribution does not differ from that of g9 (as shown in figure 7, panel B). We also introduced the GFP-U_S_9 chimeric gene from g9 in the viral genome to replace the wild type U_S_9 gene, and the resulting recombinant virus expressing GFP-U_S_9 was named Fg9.

Confocal microscopy studies in RNs infected with Fg9 and transfected with r9 were performed to analyze the distribution of plasmid-driven (red) and viral (green) U_S_9 (see top left panels r9 and Fg9 in figure 5; the two images are merged in the top right panel). Most vesicles appear yellow in the merge panel, demonstrating substantial, though incomplete, colocalization of the two chimeric proteins. Higher magnifications of different regions of the cell better define the level of colocalization. At the cell body, both in the area of higher expression/accumulation and where individual vesicles can be detected, a widespread yellow signal indicates the presence of colocalized U_S_9 and double-labeled vesicles. Nevertheless, zones/vesicles individually labeled with either the red or the green fluorophore can be spotted, indicating the presence of non-colocalizing fluorescent U_S_9 (figure 5, panel 1 merge). Identical conclusions can be drawn from the magnified distal region in panels 2 (figure 5, bottom row). The presence of these vesicles is not surprising, as viral infection normally generates a multiplicity of vesicles, including *empty* transport vesicles (i.e. containing no virion), as well as *light* particles (subvirion assemblies, with tegument and envelope proteins, but no capsid). Moreover, U_S_9 is a constitutive component of the virion and consequently, in infected cells, it is also present inside the U_S_9-labeled transport vesicle. Finally, irrespective of the source of the tagged U_S_9 gene, the plasmid-encoded viral protein present in the infected cell is functionally undistinguishable from the one expressed from the viral genome.

This experimental approach based on the co-expression in the same cell of green and red U_S_9 allows canonical co-localization analysis. However, positive results shown in figure 5 are biased by the fact that both proteins (independently of their source) are exposed to the same infected molecular environment. For this reason we also compared the localization of *stand alone* and viral U_S_9 in different cells. Here we used tubulin as a “reference” molecule to better monitor U_S_9 distribution. Even in the absence of a direct interaction with U_S_9, several lines of evidence support the choice of tubulin: intact microtubular cytoskeleton is required for virus axonal spread [39]; KIF1A, a microtubule-dependent kinesin-3 motor, interacts with PRV U_S_9, in the presence of other viral factors [17]; isolated HSV-containing organelle structures are capable of efficiently bind purified microtubules in vitro (approximately 62% of the organelles bound) [37]. Finally, we show here that the depolymerizing agent Nocodazole disrupts U_S_9 localization (Figure S1).

The top left panel of figure 6 shows the already described punctuate signal clearly detectable both at the cell body and throughout neurites in a g9-transfected neuron (with cell morphology rendered in black & white in the second top panel). The two boxes in the left panels frame the regions of the cell magnified in the two top right panels, arbitrarily chosen to show U_S_9 localization at cell body and neurites extremities. A different magnification of the same cell is also present in figure S2. Besides the Golgi region, where individual vesicles cannot be detected due to U_S_9 high concentration and expression level, virus-independent U_S_9 is frequently found in close proximity to cytoskeleton microtubules, especially in distal regions.

This fluorescence distribution is basically reproduced in RNs infected with Fg9, the recombinant HSV-1(F) virus in which the U_S_9 coding sequence was replaced by the GFP-U_S_9 chimeric gene present in g9. The Fg9-infected cell in figure 6 (left bottom panel) and Figure S2 shows a very similar fluorescent pattern, with fluorescent vesicles accumulated both in perinuclear region and distributed throughout neurites. As for transfected g9 in the top panels, two representative regions were chosen (framed boxes in left two panels) and visualized at higher magnification in the two right bottom panels of figure 6. Analysis of these images clearly show the presence of U_S_9-associated vesicles and their relationship to immuno-labeled microtubules architecture, with the overall general distribution comparable to that of GFP-U_S_9 in the absence of virus shown in the top panels. Comparison in figure 6 is indirect, as plasmid- and virus-encoded U_S_9 are present in different cells – i.e. in a uninfected or infected molecular environment, respectively. Consequently, no actual co-localization can be assessed. Nevertheless, figure 6 shows the real *stand alone* phenotype of U_S_9.

Taken together, results from the experiments depicted in figures 5 and 6 indicate that the general distribution of *stand alone* and viral U_S_9 is highly comparable, with a large, major population of vesicles containing both plasmid- and virus-derived U_S_9, and the minor presence of vesicles incorporating only one of the two chimeric fluorescent proteins. All double and single labeled U_S_9 vesicles show no preferential localization and are randomly distributed along the described punctuate pattern of either infected or transfected cells, supporting the idea that U_S_9 properties do not depend upon other viral contributions.

### Localization of mutated U_S_9

The cytosolic portion of the U_S_9 protein contains an acidic domain important for viral trans-neuronal spread [14], [20]. Of the 18 residues of serine, threonine, and tyrosine scattered throughout the protein (accounting for 20% of total amino acids), two tyrosines (Y32Y33) and two serines (S34 and S36) are part of this acidic cluster. Substitutions of these residues with alanine have been shown to have an impact on virus transport and spread [18], [20]. As already mentioned, U_S_9 is heavily post-translationally modified, and enzymes responsible for phosphorylation of tyrosines and serines in the acidic cluster have been identified or postulated, *in vitro* or *in silico* [18], [27], [30], [40]. Given this background, it was of absolute relevance to investigate whether these modifications are required to the *stand alone* molecular properties of U_S_9. We constructed two new U_S_9 fluorescent chimaeras, in which tyrosines 32 and 33 or serines 34 and 36 where substituted by alanines, named g9yyaa and g9aea, respectively. The localization patterns of the two U_S_9 mutants were analyzed and compared in figure 7 with the RFP-U_S_9 wt fluorescent staining. RNs contransfected as outlined showed the typical vesicles distribution already described. Both g9yyaa (figure 7C) and g9aea (figure 7D) localize in puncta scattered throughout neurites and are also present in an intensively fluorescent area surrounding the nuclei. Merging the images obtained by wild type (red) and mutants (green) U_S_9 localization generates a perfect yellow pattern (figure 7C and 7D, right panels). From these results we can conclude that these U_S_9 modifications, shown to affect virus transport and infection spread in PRV models, do not modify U_S_9 behavior, supporting the hypothesis that U_S_9 does not require additional viral factors in order to exert its transport function.

## Discussion

Efficient and specific transport of single molecules and multi-molecular complexes to different cellular compartments and structures or to the outside environment is a key feature of eukaryotic cells. Viruses - unwanted travelers that must efficiently move inside the infected cell in order to reach specific compartments and complete their replication cycle [41] - use the highly efficient and specialized cell transport system. Identification and analysis of viral proteins contributing to (or determining) this dynamic phenotype can eventually lead to a virus-independent approach to control cellular transport and delivery. From this perspective we looked at U_S_9, a viral protein highly conserved amongst alphaherpesviruses, with a critical role in anterograde spread of the virus [7]–[12], [19]. Herpes Simplex Virus is a lifelong guest of infected hosts, in which it becomes and remains latent in dorsal root ganglion sensory neurons [42]. Given the peculiar replication cycle of the virus and the morphology of the infected neurons, HSV must traverse very long distances inside the cell, in both retro- and antero-grade directions, making it a good potential tool to understand (and conceivably direct) cellular transport machinery while gaining insides into viral trafficking. Deletion of U_S_9 gene from the genomes of several alphaherpesviruses generates recombinant viruses with impairments in the ability to spread in the nervous system due to defects in anterograde transport [8]–[12], [15], [23]. Studies aimed at defining the intracellular localization of the viral protein showed a punctuate staining in cells expressing U_S_9 fused to GFP, with a steady-state accumulation in the TGN region and vesicles scattered throughout the cell [8]. U_S_9 was also found at the plasma membrane, from which it is recycled into cytoplasmic vesicles [20].

The key question we sought to address here is whether HSV U_S_9 maintains its transport properties in the absence of other viral factors, and independently of its natural cargo, i.e. the virion. Our long-term goal is to use this information to be able to exploit Us9 as tool for directed cellular/intercellular delivery, in the context of both mechanistic studies and drug-delivery purposes. In infected cells, besides being a constitutive component of transported virions, U_S_9 is present on the membrane of transport vesicles. We reasoned that for a viral protein to be necessary for virus transport, it must be part of the molecular complex that connects the cellular transport machinery to its cargo. This implies that U_S_9 (as any other protein with similar function) exerts two, possibly independent, activities. We may call these *transport activity* (i.e. the ability to engage cellular transport machinery and become part of vesicles that are exported along cytoskeleton structures) and *cargo selection* (i.e. the ability to direct virion load and consequent transport). Our study has been instrumental in determining the extent to which these two functions are independent. This is a necessary condition to establish U_S_9 as an autonomous transport protein for trafficking studies or specific delivery.

HSV-1 GFP-U_S_9 chimera constructed here shows the distinctive fluorescent pattern in transfected cells, i.e. a bright punctuate signal indicating a vesicular expression, with labeled vesicles scattered throughout the cytoplasm and partially concentrated in a perinuclear region already defined as the steady-state site of accumulation of U_S_9, and identified as Trans Golgi Network [8]. Additionally, U_S_9 is detectable at the plasma membrane. GFP-U_S_9 localization is almost identical in all tested cell types, suggesting that its behavior is an intrinsic property of the protein. Also interesting is the finding that U_S_9-containing vesicles are always found in peripheral regions. In cells with elongated shape U_S_9 is spotted at cellular tips, and this distal staining is not directly related to the intensity of the signal in the perinuclear region.

The key evidence for the U_S_9 autonomous transport properties comes from real time experiments in which transfected cells are imaged *in vivo*, and GFP-U_S_9 localization is recorded over an extended period of time. The resulting movie shows the actual highly dynamic behavior of U_S_9 in the absence of other viral factors. Focusing on elongated regions of the cell (though the phenotype is not restricted to these areas), fluorescent vesicles can be seen traveling in both antero- and retrograde directions. Some vesicles invert the direction they move over the period the movement is recorded. This discontinuous activity is not surprising as often vesicles moving in one direction can apparently invert their motion by jumping on near microtubules tracks or through the coordination activity of a macromolecular complex [43]–[46]. As a consequence, the final transport may result from several short movements that can occur in both directions.

In PRV, U_S_9 has been shown to interact with KIF1A, a microtubule-dependent kinesin-3 motor, and the interaction is dependent on the presence of other still unidentified viral factor(s) [17]. The same report demonstrates that HSV-1 U_S_9 cannot be co-purified with KIF1A, launching the quest for a HSV U_S_9 motor partner. Irrespective of the identity of that molecule, our data suggest that HSV U_S_9 can engage the cellular transport machinery, and that its dynamic behavior occurs in the absence of other viral factors. It is possible that U_S_9-dependent axonal transport of HSV particles requires additional proteins, as suggested by the PRV data, with glycoproteins E and I being good, but not exclusive, candidates to play such a role. Nevertheless and not considering virion transport, U_S_9-containing vesicles traffic occurs with no additional factors.

In primary neurons, the GFP-U_S_9 localization could be clearly appreciated and may result extremely helpful to understand the molecular basis of U_S_9 activity. In these cells (as on the other cell types analyzed), GFP-U_S_9 fluorescent vesicles localize both at cell body and throughout neurites, frequently in close proximity to microtubules (figure 6, g9). Identical distribution and microtubules proximity (in distal regions) were seen in neurons infected with a recombinant virus expressing GFP-U_S_9 (figure 6, Fg9), showing no differences with the behavior of the viral protein expressed in the absence of other viral factors. By using different fluorescent tags to distinguish *stand alone*- (transfected), and virus-encoded-U_S_9, we were able to precisely assess the extent of co-localization. The two differently tagged versions of U_S_9 co-expressed in the same cell mostly localize in the same vesicles, in both proximal and distal cellular regions. Few but constantly present individually tagged vesicles with no preferential sub-localization were detected in all the experiments, and this reproducible distribution can be interpreted with the equivalence between the two differently tagged U_S_9. As well established, due to the peculiar way viruses replicate themselves, there is no difference between *stand alone* (transfected) RFP-U_S_9 and virus-encoded GFP-U_S_9, once they are co-expressed in the same infected cell. The presence of few green or red, and many yellow vesicles simply demonstrates U_S_9 incorporation in these transport vesicles (not all necessarily carrying complete viral particles, as natural infection always produces empty and light particles).

In agreement with our hypothesis of an autonomous transport activity is the observation that ablations of two phosphorylation sites in the cytosolic tail of U_S_9 do not affect U_S_9 localization. PRV transneuronal spread depends (to different extents) on the presence of two tyrosines and two serines in a conserved acidic cluster [18], [20]. Results presented here, showing co-localization of wild type U_S_9 with both mutant proteins, indirectly support the idea that no other viral factors are required for the U_S_9 transport function. This interpretation implies that U_S_9 is able to autonomously traffic inside the cells, while other modifications and/or viral factors are required to make the virus (or its parts) be transported along. In other words, viral or cellular modifications of U_S_9 may affect cargo loading specificity and not U_S_9 transport activity. As a consequence, U_S_9 may travel even in the absence of other viral functions that are required for virus transport (i.e. acidic domain modifications).

In this study, GFP was used as a tool to chase U_S_9 localization. However, from a different standpoint, GFP may be also seen as a molecule targeted to specific destinations by the addition of the U_S_9 sequence. Importantly, this U_S_9 sequence is sufficient to confer the resulting GFP fusion protein the specific localization pattern described, even though GFP is about three times larger than full length U_S_9. Thus U_S_9 is not only a valid molecular tool for the study of axonal transport but may also be used to deliver large proteins or other targets to specific neuronal sites. To achieve this goal, a full understanding of how U_S_9 transport vesicles are formed and loaded is necessary. Once revealed, these mechanisms may be exploited to accomplish transport delivery and design/test different loading strategies.

## Materials and Methods

### Ethics Statement

Animals were used as a source of brain tissue to prepare neuronal cultures, following the recommendations in the Guide for the Care and Use of laboratory Animals of the National Institute of Health. This protocol for harvesting brain tissue was approved by the Institutional Animal Care and Use Committee of Drexel University (PHS Animals Welfare Assurance #A-3222-01), approved on 10-18-2012 (permit #20098).

### Cells, transfections, infections and treatments

MDA-MB-231 is a human breast cancer cell line [47]. HOS is a hosteosarcoma cell line of human origin, with mixed, fibroblast and epithelial like morphology, (obtained through the NIH AIDS Reagent Program, Division of AIDS, NIAID, NIH: HOS.p.BABE-puro from Dr. Nathaniel Landau [48]–[49]). Saos-2 is a non-transformed cell line derived from the primary osteosarcoma of an 11-year-old Caucasian girl [50]. HEp2 cells [51] were derived via HeLa contamination. Vero cell line [51] was derived from kidney of a normal adult African green monkey. All cells lines were cultured in Dulbecco Modified Eagle Medium (DMEM) supplemented with 10% Fetal Calf Serum. Normal human astrocytes (HA) derived from neural precursor cells were purchased from ScienCell Research Laboratories (San Diego, CA), and cultured in Astrocyte Medium supplemented with fetal bovine serum (2%) and Astrocyte Growth Supplement provided by the vendor. Rat cortical neurons (RNs) were obtained from the brains of 17–18 day old rat embryos and cultured in Neurobasal medium containing B27 supplement as detailed by Sengupta et al. [52] and originally described by Brewer et al. [53].

For transfections, cells were seeded on 35 mm dishes or on glass coverslips in 35 mm dishes 16–24 hours prior to the experiment. RNs were plated on poly-L-lysine-coated coverslips. Transfections were performed with Lipofectamine 2000 (Invitrogen), using 2 µg of plasmid DNA. In all the experiments described, plasmids were introduced into the cells by transient transfection, and routinely ≥50% of transfected cells were obtained, as estimated by fluorescence microscopy. Nocodazole was obtained from Sigma, and used at 1 µg/ml concentration.

Herpes Simplex Virus 1 (HSV-1) strain F, and the derived mutant strain Fg9 were amplified and titered in Vero cells, and inoculated in RNs at multiplicity of infection (MOI) = 10 plaques forming units per cell (pfu/cell).

### Plasmids and recombinant viruses construction

The U_S_9 sequence was amplified from HSV-1 genome and cloned into pEGFP-C1 (Clontech) to generate g9, with the insertion of the Hemoagglutinine (HA) epitope. The intercalary spacer between the two molecules results in the following peptide: KSGLRSISSSSFEFMAYPYDVPDYASLGGHMAMGMTS ( EGFP - HA - U_S_9).

To generate a red fluorescent version of tagged U_S_9, the GFP sequence in g9 was substituted with the RFP sequence from pTagRFP-tubulin (Evrogen, Moscow. Russia).

g9-ΔTM was obtained by collapsing in g9 the trans-membrane sequence comprised between two SalI sites, present in the middle of the sequence encoding the 6 arginine stretch of U_S_9 (figure 1A) and in the multiple cloning site of pEGFP-C1. g9-ΔTM has the same topological orientation as g9, with the U_S_9 sequence lacking the trans-membrane coding portion.

GFP fused to the U_S_9 trans-membrane domain was obtained by inserting the U_S_9 sequence starting from SalI site in the middle of the 6 arginine stretch (figure 1A) into pEGFP-C1 XhoI site. The resulting plasmid was called g9-TM and lacks the HA epitope.

g9yyaa and g9aea are mutant versions of g9 in which U_S_9 codons TAC-TAC (encoding two tyrosines at positions 32 and 33) and TCG-GAA-AGC (encoding serine-glutamic acid-serine at positions 34–36) have been mutagenized to obtain GCC-GCC (alanine-alanine) and GCG-GAA-GCG (alanine-glutamic acid-alanine), respectively.

To obtain the recombinant HSV-1 expressing the GFP-U_S_9 protein, the chimeric g9 coding sequence from g9 plasmid was cloned into an intermediate vector containing the BamHI X fragment from the HSV genome, with a short polylinker replacing the U_S_9 sequence downstream of the gene sixth codon. In the resulting plasmid, GFP-U_S_9 fusion gene substitutes the original U_S_9 sequence, with large enough viral genomic homologous regions on both sides of the insertion. This plasmid was co-transfected in Vero cells with the viral genomic DNA and recombinant viruses in fluorescent plaques were isolated from the monolayer. The recombinant virus, named Fg9, was plaque-purified for 5 times and finally titered in Vero cells. The resulting viral fusion protein expression is under U_S_9 natural promoter control and in the same genetic context, as GFP-U_S_9 translation begins after the first 6 U_S_9 aminoacids.

All plasmids generated in the study were sequenced.

### Protein electrophoretic analysis and western blotting

Protein extracts were separated on denaturing polyacrylamide gels (SDS Page) and transferred to a PVDF membrane for immunoblotting. Cell fractionation was performed as described by Sadoul et al. [33] and Fournier et al. [34]; post-nuclear fractions containing soluble (S) or crude membrane (M) proteins were analyzed by SDS Page. Primary antibodies used were the U_S_9 specific antibody [31], and anti-GFP antibody (sc-8334, Santacruz Biotechonology). Secondary antibodies and detection reagents were from Pierce (SuperSignal West Femto kit).

### Fluorescence microscopy analysis and acquisition

Except for real time experiments, cells were fixed in 4% paraformaldehyde at the indicated times post transfection/infection. For cytoskeleton immuno-staining of RNs, fixed cells were permabilized in Phosphate Buffered Saline (PBS) containing 0.2% Triton, 1% Bovine Serum Albumine (BSA), 0.5% Newborn Calf Serum (NCS) for 30 minutes at Room Temperature. Permeabilized cells were first incubated with a monoclonal antibody against neuronal class III β-Tubulin, clone TUJ1 (Covance) and then with the anti-mouse Alexa Fluor 568 from Life Technologies. For MDA cells, antibodies used were anti β-tubulin (Sigma, T4026) and anti-mouse IgG (Fab specific)–TRITC (Sigma, T7782). Nuclear counter-staining was done via incubation of fixed cells with Hoechst 33342 (Molecular Probes - Invitrogen).

Confocal images were taken under a Zeiss Axio Imager.Z1m microscope equipped with Plan-apochromat 63×/1.4 oil DIC objective. For all the other experiments, cells were observed and recorded under the Nikon Eclipse E600 fluorescence microscope (filters UV2A, B2A, G2A) equipped with 100× objective. Acquisition software was the provided Nikon ACT-2U. For real time imaging, the output generated by ACT-2U was captured by the freeware Debut. All images were analyzed and assembled using ImageJ. Graphic elements were added using Gimp2 and Inkscape. Images are representative of the original data and comply with the journal image manipulation policy.

## Supporting Information

Figure S1 **U_S_9 localization is dependent on the integrity of cytoskeleton.** Intracellular trafficking is organized along cytoskeletal structures. Cells treated with Nocodazole, an antimitotic agent commonly used to disrupt the cellular cytoskeleton, undergo a complete rearrangement of cytoskeleton, with tubulin molecules becoming redistributed throughout the cytoplasm. Nocodazole effect is reversible, as incubation of treated cells in medium lacking the drug leads to a complete recovery of normal cellular cytoskeletal structures. MDA cells transfected with g9 plasmid were treated for the indicated time (0, 30, 60, and 120 minutes) with Nocodazole, immunostained with an anti-tubulin antibody, and imaged under a fluorescence microscope. Effect of treatment on U_S_9 localization (in green) is compared to the disruption of cytoskeleton (immunostained in red with an anti-tubulin antibody) induced by the presence of the drug. The complete rearrangement of U_S_9-associated vesicular staining (g9 t30 through g9 t120) increases in a time dependent manner and perfectly matches tubulin disorganization. 30 minutes after removal of Nocodazole from culturing medium (g9 t120 rec) cytoskeleton structures begin to appear, and corresponding U_S_9-labeled normal vesicular pattern becomes detectable (white arrows in panel ‘g9 t120 rec’). 2 hours nocodazole treatment of cells expressing g9-TM truncated form does not seem to have a major impact on the chimera localization. Bar = 10 µm.
(TIF)

Figure S2 **Localization in neurons of U_S_9 expressed from the viral genome or in virus-free context.** RNs were either transfected with the GFP-U_S_9 plasmid (g9: top panels) or infected (MOI = 10 pfu/cell) with the recombinant HSV-1(F) carrying the chimeric GFP-U_S_9 gene (Fg9: bottom panels). Left first panels in both rows show the bright fluorescent punctuate pattern in transfected or infected cells. Central panels show the immunostained microtubular cytoskeleton. In right panels, the two images on the left have been merged. Localization of transfected U_S_9 seems to mostly overlap that of U_S_9 expressed in the viral context. This supplemental figure shows a different magnification of the same cells in figure 6. Bar = 10 µm.
(TIF)

Movie S1 **U_S_9 trafficking does not depend on other viral functions.** Real time imaging of GFP-U_S_9 vesicles trafficking inside a MDA transfected cell. Only a portion of a g9-transfected MDA cell is shown in the movie.
(AVI)

Movie S2 **Directional transport of U_S_9 vesicles.** Movie showing a portion of a GFP-U_S_9-transfected MDA cell imaged in real time. Frames from Movie S1 have been chosen and labeled with green and red arrows to track the movements of arbitrary chosen vesicles. Green arrows = anterograde direction; red arrows = retrograde direction.
(AVI)
