# Supplementary figures and images for: Molecular Features Contributing to Virus-Independent Intracellular Localization and Dynamic Behavior of the Herpesvirus Transport Protein US9
Source: PLoS One. 2014 Aug 18;9(8):e104634. doi: 10.1371/journal.pone.0104634 (PMC4136771; doi:10.1371/journal.pone.0104634)

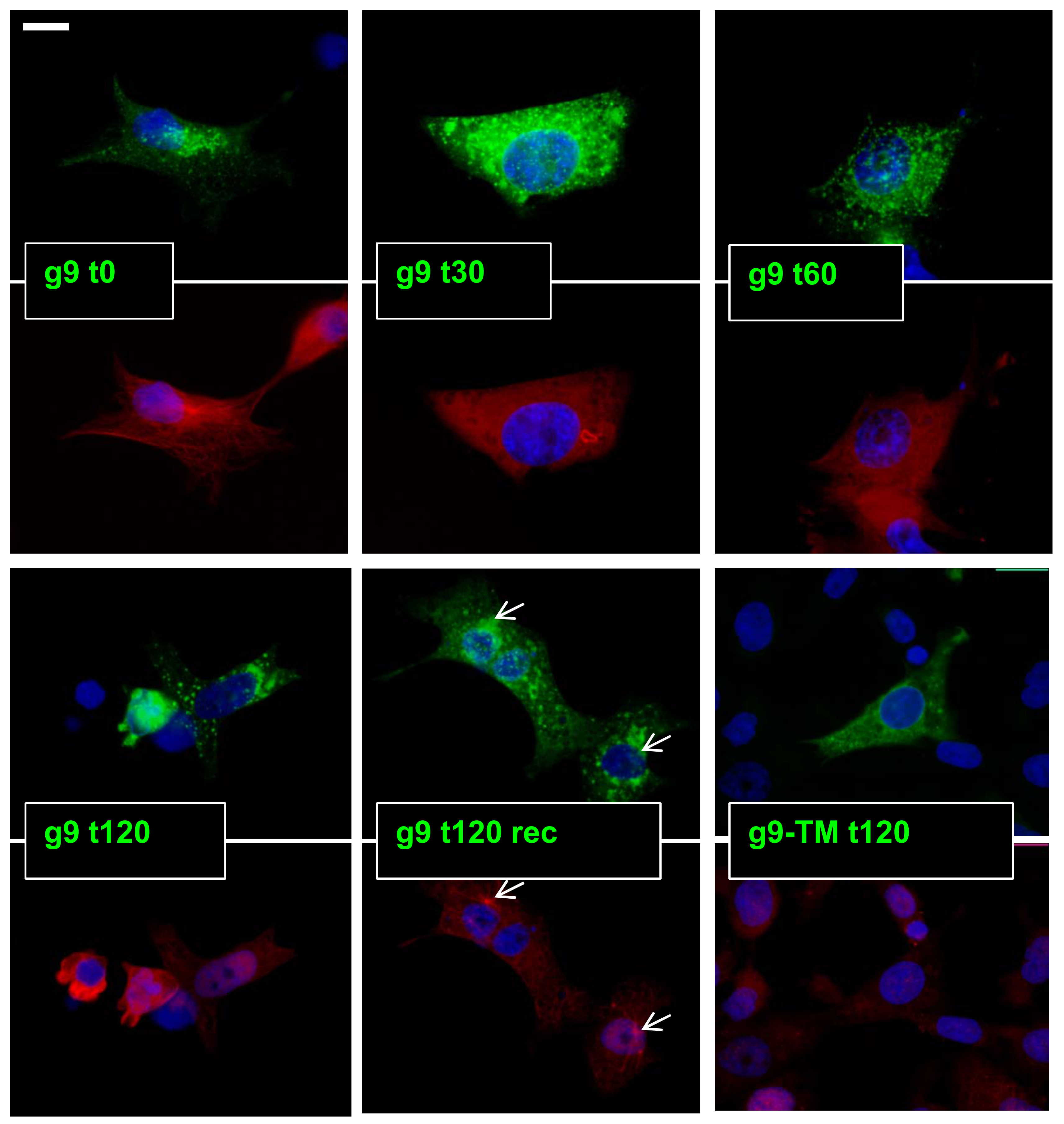

Supplement: Figure S1 — US9 localization is dependent on the integrity of cytoskeleton. Intracellular trafficking is organized along cytoskeletal structures. Cells treated with Nocodazole, an antimitotic agent commonly used to disrupt the cellular cytoskeleton, undergo a complete rearrangement of cytoskeleton, with tubulin molecules becoming redistributed throughout the cytoplasm. Nocodazole effect is reversible, as incubation of treated cells in medium lacking the drug leads to a complete recovery of normal cellular cytoskeletal structures. MDA cells transfected with g9 plasmid were treated for the indicated time (0, 30, 60, and 120 minutes) with Nocodazole, immunostained with an anti-tubulin antibody, and imaged under a fluorescence microscope. Effect of treatment on US9 localization (in green) is compared to the disruption of cytoskeleton (immunostained in red with an anti-tubulin antibody) induced by the presence of the drug. The complete rearrangement of US9-associated vesicular staining (g9 t30 through g9 t120) increases in a time dependent manner and perfectly matches tubulin disorganization. 30 minutes after removal of Nocodazole from culturing medium (g9 t120 rec) cytoskeleton structures begin to appear, and corresponding US9-labeled normal vesicular pattern becomes detectable (white arrows in panel ‘g9 t120 rec’). 2 hours nocodazole treatment of cells expressing g9-TM truncated form does not seem to have a major impact on the chimera localization. Bar = 10 µm. (TIF) [file pone.0104634.s001.tif]

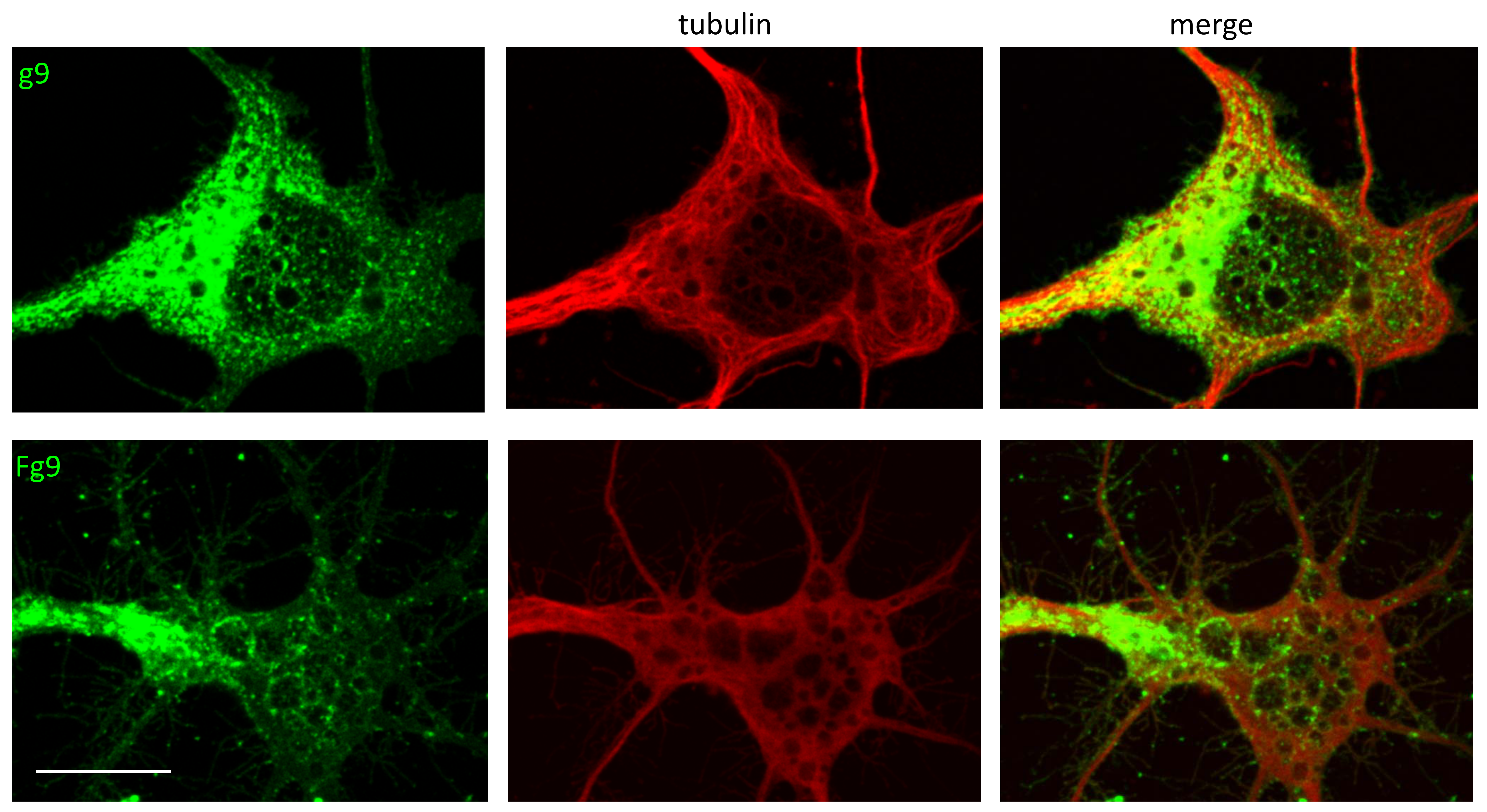

Supplement: Figure S2 — Localization in neurons of US9 expressed from the viral genome or in virus-free context. RNs were either transfected with the GFP-US9 plasmid (g9: top panels) or infected (MOI = 10 pfu/cell) with the recombinant HSV-1(F) carrying the chimeric GFP-US9 gene (Fg9: bottom panels). Left first panels in both rows show the bright fluorescent punctuate pattern in transfected or infected cells. Central panels show the immunostained microtubular cytoskeleton. In right panels, the two images on the left have been merged. Localization of transfected US9 seems to mostly overlap that of US9 expressed in the viral context. This supplemental figure shows a different magnification of the same cells in figure 6. Bar = 10 µm. (TIF) [file pone.0104634.s002.tif]
